# Effect of Permeable Crystalline Materials on the Mechanical and Porosity Property of Recycled Aggregate and Recycled Aggregate Concrete

**DOI:** 10.3390/ma16134596

**Published:** 2023-06-26

**Authors:** Pengfei Li, Wenhao Gan, Guoyou Yao, Qiao Huang, Renming Zhao

**Affiliations:** 1 College of River and Ocean Engineering, Chongqing Jiaotong University, Chongqing 400074, China; 622200960068@mails.ctjtu.edu.cn (W.G.); 622210090018@mails.cqjtu.edu.cn (Q.H.);; 2 Suzhou Guardex New Material Technology Co., Ltd., Suzhou 210500, China; yaoguoyou@rc-guardex.com

**Keywords:** recycled aggregate concrete, permeable crystalline materials, compressive strength, microstructure, mechanism of modification

## Abstract

This study investigates the potential of permeable crystalline materials to improve the properties of recycled aggregates and recycled aggregate concrete (RAC). The use of recycled aggregates in concrete production has gained increasing attention due to environmental and economic benefits. However, the lower quality and poorer durability of recycled aggregates limit their wider application. In this study, three types of recycled aggregates were treated with permeable crystalline materials, and their water absorption and crushing index were compared before and after modification. RAC was then produced using modified recycled aggregates with different substitution rates, and their mechanical properties were evaluated. To investigate the mechanism of permeable crystalline materials modification, the microstructure of the modified RAC was observed using nuclear magnetic resonance and scanning electron microscopy. The results demonstrated that the permeable crystalline materials treatment effectively reduced the water absorption and crushing index of the recycled aggregates. The compressive strength of modified RAC also improved, with a higher modification time leading to higher strength. Furthermore, the pore distribution and microstructural denseness of the modified recycled aggregates and RAC were enhanced, as revealed by the microstructural observations. These findings suggest that permeable crystalline materials modification is a promising method for improving the properties of recycled aggregates and RAC, which could contribute to the sustainable development of the construction industry.

## 1. Introduction

Nowadays, with the rapid growth of population and economy, the need for renovating old buildings and reconstructing infrastructure results in a large amount of construction waste [1]. Discarded concrete constitutes a significant portion of this waste. However, the traditional approach to handling construction waste is environmentally harmful and fails to realize the potential value of discarded concrete [2]. Therefore, an effective solution is needed to handle construction waste and increase the utilization of discarded concrete.

One such solution is recycling concrete waste by converting it into recycled aggregate (RA). Waste concrete can be crushed and screened to produce RA, which can be used as a substitute for natural aggregates [3]. However, RA possesses properties such as low strength and high water absorption, which limit its practical engineering applications [4,5,6]. Research indicates that the mechanical properties of concrete depend on the strength of the interfacial transition zone (ITZ) of concrete [7,8]. In recycled aggregate concrete, the presence of a higher quantity of poor quality ITZs results in lower mechanical properties [9,10]. Perdro [11] prepared recycled aggregate concrete using 100% aggregate replacement rate using three different initial strengths of recycled aggregates. It was found that the initial strength of the aggregates had an effect on the performance of the recycled aggregate concrete. Job [12] prepared recycled aggregate concrete using different aggregates and unused aggregate replacement rates. It was found that the highest substitution rate was up to 25%, and the concrete performance decreased significantly as the aggregate substitution rate increased.

In order to improve the performance of recycled aggregates, many scholars have attempted various methods. Some have tried to remove old mortar from recycled aggregates using mechanical grinding, heat treatment [13,14,15], and chemical treatment [16]. However, these methods lead to increased energy consumption, carbon dioxide emissions, and waste powder generation. Others have used reinforcement carbonation and microbial carbonate precipitation methods to precipitate calcium carbonate in old mortars of recycled aggregate, but they could introduce alkali and increase the risk of concrete damage [17,18,19]. Nanomaterial modification technology has also been explored for modifying recycled aggregate concrete [20,21]. By adding nanomaterials [22,23] to recycled aggregate concrete to modify the properties of fresh concrete, the mechanical properties and durability of concrete can be improved [24,25]. However, the uneven distribution of particles in the cement matrix affects the improvement effect [26].

This study proposes the use of permeable crystalline materials to modify recycled aggregates and improve the properties of recycled aggregate concrete (RAC). Given the variables of recycled aggregates with varying initial strengths, modification times, and replacement rates, the effects of permeable crystalline materials on the performance indicators of recycled aggregates and the strength and microstructure of RAC were studied. The aim of this research is to explore the enhancement of permeable crystalline materials on the multi-scale performance of RAC, providing a more sustainable and efficient solution for handling construction waste and increasing the utilization of recycled concrete.

## 2. Materials and Methods

### 2.1. Materials

#### 2.1.1. Raw Materials

**Figure 1:**
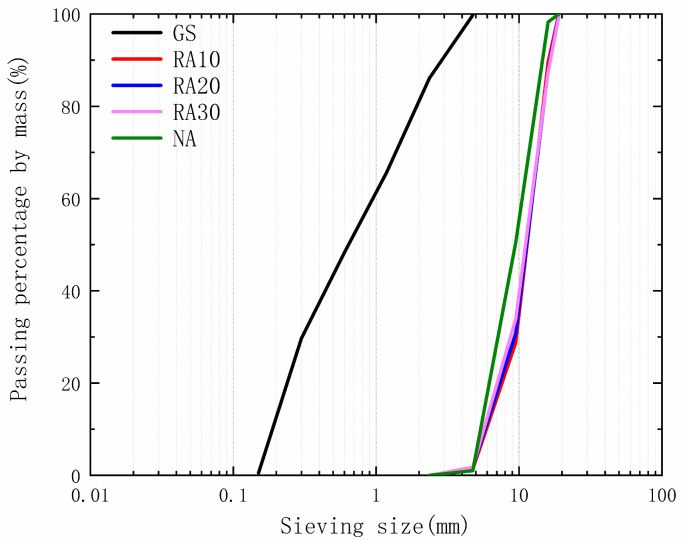
Sieving curves of various aggregates.

The study utilized various raw materials, including Portland cement with a strength class of 52.5 N, first grade fly ash, quartz sand, limestone, and a polycarboxylate superplasticizer. The cement had a density of 3.21 g/cm^3^, and its chemical composition is presented in Table 1. The FA used was also grade I with a density of 2.55 g/cm^3^, and its chemical composition is listed in Table 1 as well. The fine aggregate was composed of quartz sand with a particle size ranging from 0.075 mm to 4.75 mm and a density of 2.73 g/cm^3^, as shown in Figure 1. Limestone was used as the natural aggregate, and its physical properties are presented in Figure 1 and Table 2. Additionally, a polycarboxylate superplasticizer with an apparent density of 1.20 g/cm^3^ was used as an additive.

#### 2.1.2. Permeable Crystalline Materials

**Figure 2:**
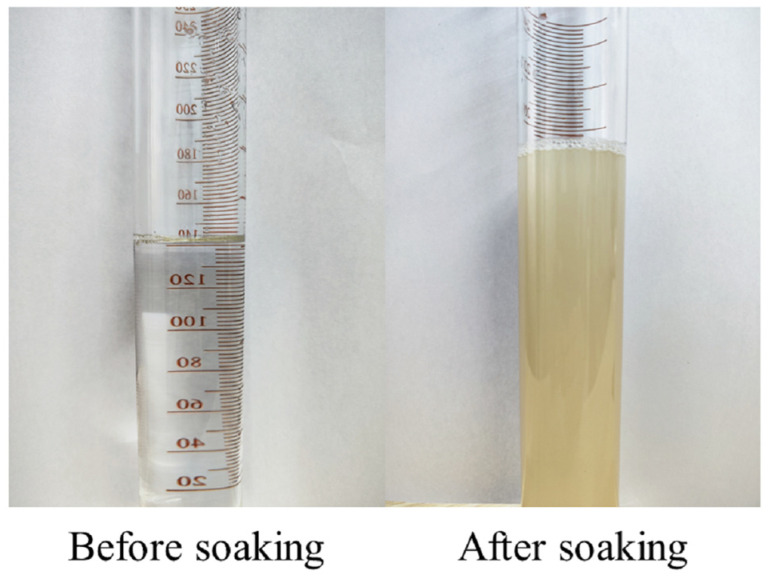
Permeable crystalline materials.

The study employed permeable crystalline materials provided by Suzhou Guardex New Material Technology Co., Ltd. (Suzhou, China). These materials are primarily composed of silica ions, which are highly soluble in water, and are presented in the form of a transparent and colorless liquid, as depicted in Figure 2. To treat the recycled aggregates, they were directly immersed in the permeable crystalline solution.

#### 2.1.3. Recycled Aggregates

**Figure 3:**
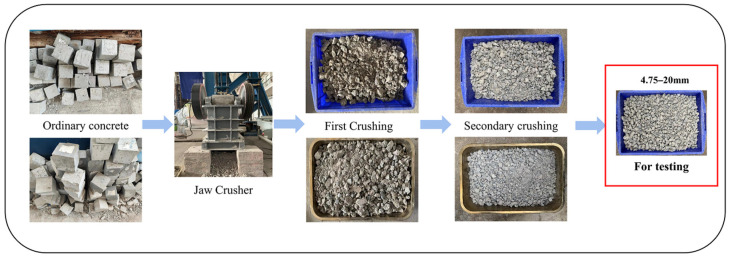
Preparation process of recycled aggregates.

The recycled aggregates used in this study were laboratory-prepared ordinary concrete. After 28 days of curing, the compressive strengths of these ordinary concretes were 32.6 MPa, 25.5 MPa, and 19.7 MPa, respectively, and were designated as C30, C20, and C10, respectively. After the curing period, the concrete was crushed, sieved, cleaned, and air-dried to obtain the recycled aggregates. The recycled aggregates prepared from the concrete were designated as RA30, RA20, and RA10, respectively. The resulting aggregates were then separated into two size ranges of 4.75–10 mm and 10–20 mm for further modification and physical property testing. The production process of the recycled aggregates is illustrated in Figure 3. The physical properties of the recycled aggregates are presented in Figure 1 and Table 2.

### 2.2. Sample Preparation

To modify the recycled aggregate, the following procedure was implemented:

(1) The recycled aggregate was immersed in the permeable crystalline solution for 14, 21, and 35 days, respectively.
(2) Upon reaching the target immersion time, the recycled aggregate was removed from the permeable crystalline solution.
(3) The removed recycled aggregate was washed and air-dried.
(4) The modified recycled aggregates were subjected to tests to determine the crushing index and water absorption rate.

**Figure 4:**
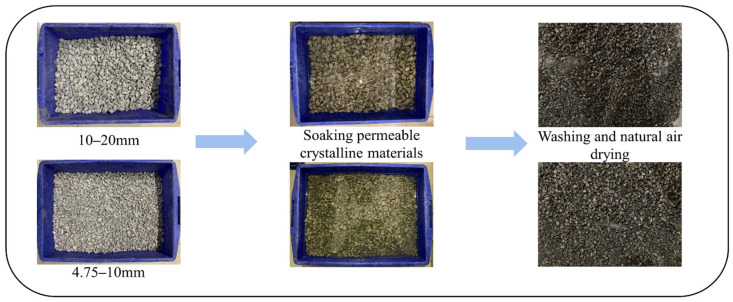
Modification process of recycled aggregate.

The modification process of the recycled aggregate is depicted in Figure 4.

### 2.3. Mix Proportion Design

To mitigate the effects of water absorption, the recycled aggregates underwent pre-wetting with water and were saturated for an hour prior to mixing with the concrete. The experiment was designed with 25 sets of proportions, and 6 concrete test blocks were cast for each set of proportions. The concrete specimens were cured in the laboratory for 24 h and then transferred to a standard curing chamber for an additional 28 days. The concrete was designed for a strength of C50, with a water–cement ratio of 1:1, conforming to the specifications for self-compacting concrete design. Recycled aggregates were used to replace natural aggregates in equal masses, with replacement rates of 0%, 50%, and 100%, using particle sizes of 4.75–10 mm and 10–20 mm, in a 6:4 ratio by mass. Moreover, a comparative test was conducted to examine the enhancement in mechanical properties of recycled aggregate concrete by modifying it with permeable crystalline materials at different replacement rates of aggregates before and after modification. The mix proportion of modified recycled aggregate concrete is presented in Table 3.

### 2.4. Testing Methods

#### 2.4.1. Crushing Indices of Recycled Aggregates

The crushing index of the recycled aggregates was determined in accordance with the construction test standard for pebbles and crushed stones (GB-T14685-2011) [27]. First, a 3000 g specimen was loaded into a round mold in 2 layers and then subjected to 25 cycles of alternate reversal. The mold was then covered with an indenter after leveling. Next, the sample was placed on a press and loaded uniformly to 200 kN at a rate of 1 kN/s. After a 5 s stabilization period, the sample was unloaded. Finally, the crushed samples were sieved with a sieve having a size below 2.36 mm. The crushing index was calculated by dividing the mass of the sample on the screen by the total mass of the sample. To ensure accuracy and consistency, this test was repeated multiple times for each recycled aggregate sample, and the average value of the crushing index was calculated for each size range of the recycled aggregates.

#### 2.4.2. Water Absorptions of Recycled Aggregates

The water absorption test was carried out in accordance with the standard test for pebble and crushed stone used in construction (GB-T14685-2011). To avoid any potential damage to the internal structure of the recycled aggregate due to high-temperature drying and to minimize experimental error, the water absorption of the aggregate was tested using the natural air-drying method for 72 h. After the test, the samples of recycled and modified recycled aggregates were discarded directly to reduce the error caused by sampling pairs.

#### 2.4.3. Flowability Testing of Recycled Aggregate Concrete

The properties of the recycled aggregate self-compacting concrete were evaluated through two standard tests: the V-funnel test and the concrete slump test. The concrete slump test involves filling a bucket with concrete and measuring the resulting “slump” after the bucket is lifted to determine the concrete’s flowability and workability. Conversely, the V-funnel test measures the time required for the concrete to flow out of a specially designed funnel and is particularly useful for assessing the viscosity and flowability of self-compacting concrete. After mixing the recycled aggregate self-compacting concrete in the laboratory mixer, both tests were conducted following standard specifications.

#### 2.4.4. Compressive Strength of Recycled Aggregate Concrete

The compressive strength of the concrete was measured in accordance with the Standard Test Method for Performance of Ordinary Fresh Concrete (GB/T50080-2016) [28]. The specimens were cured under standard conditions of 20 ± 2 °C and 95% humidity for 28 days before testing. For each mixture, three concrete specimens were prepared to measure the compressive strength. The loading rate was fixed at 0.6 MPa/s to ensure accuracy and consistency.

#### 2.4.5. Nuclear Magnetic Resonance Test

Nuclear magnetic resonance (NMR) tests were conducted to investigate the effect of permeable crystalline materials on the microstructure and internal void distribution of recycled aggregates (RA) and recycled aggregate concrete (RAC). The following test procedures were conducted: (1) Standard cube specimens were prepared and cured for 28 days, after which 3 cylindrical specimens measuring 25 mm in diameter and 40 mm in height were cut from each cube for testing. (2) The specimens were then vacuum-saturated for 24 h to ensure complete saturation. (3) The pore size distribution and porosity of the saturated specimens were analyzed using a Newman core analyzer.

#### 2.4.6. Scanning Electron Microscope Test

The scanning electron microscope (SEM) analysis was conducted using an FEI QUANTA FEG250 (FEI Corporation, Hillsboro, OR, USA) thermal field emission scanning electron microscope. A 10 mm × 10 mm × 10 mm block was selected for testing, and the specimens were prepared by water stopping, followed by drying to a constant weight and gold coating. SEM analysis was performed after the compressive strength test to investigate the microstructure of the recycled aggregate concrete and the impact of the modification process on the microstructure.

## 3. Results

### 3.1. Effect of Permeable Crystalline Materials on the Crushing Index of Recycled Aggregate

**Figure 5:**
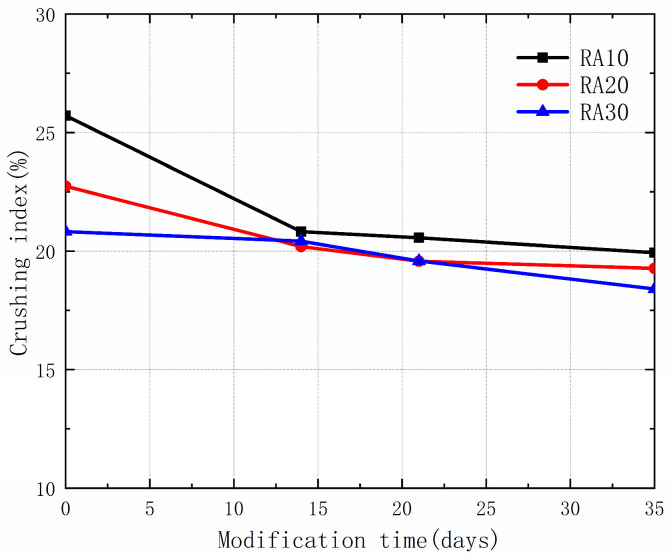
Crushing index of RA and MRA.

The crushing index is a crucial parameter for evaluating the mechanical performance of aggregates. The effect of permeable crystalline materials on the crushing index of recycled aggregates was investigated in this study. Figure 5 shows the crushing index of all modified recycled aggregates. The results indicate that the crushing index of recycled aggregates decreased after modification with permeable crystalline materials. Further analysis showed that the greatest decrease in the crushing index of the three types of aggregates occurred when the modification time was 35 days (22.45% decrease), followed by 21 days (20.00% decrease), and then 14 days (18.99% decrease). This suggests that the improvement effect on the crushing index of recycled aggregates becomes better with longer modification time. The permeable crystalline materials infiltrate into the pores of the recycled aggregates along with water, reacting with strongly oxidized calcium to produce crystalline compounds, thereby enhancing the strength of the recycled aggregates [29]. However, it should be noted that the efficiency of improving the crushing index of recycled aggregates decreases as the initial strength of the recycled aggregates increases. Therefore, for recycled aggregates with high initial strength, alternative approaches should be considered to modify their aggregate properties.

### 3.2. Effect of Permeable Crystalline Materials on the Water Absorption of Recycled Aggregate

**Figure 6:**
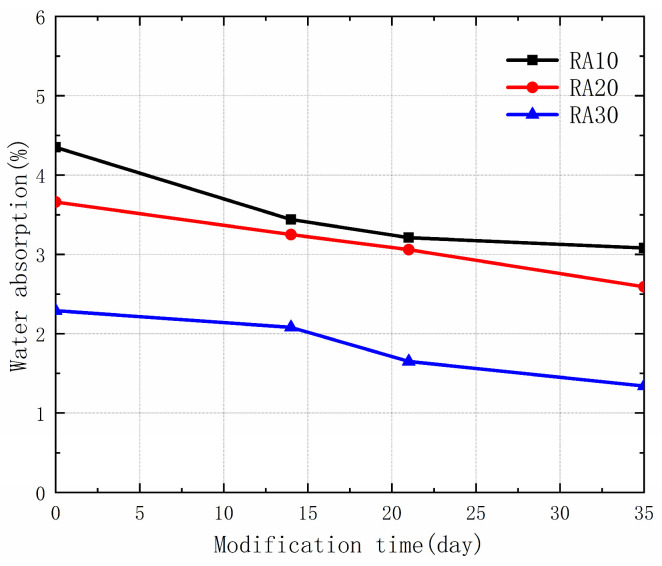
Water absorption of RA and MRA.

Water absorption is an important parameter for evaluating the quality of recycled aggregates, as it can affect the durability and long-term performance of concrete. Figure 6 shows the water absorption of different types of recycled aggregates under different modification times using permeable crystalline materials. The results indicate that the water absorption of the recycled aggregates decreased after modification, with the improvement becoming more significant with longer modification times. The maximum reduction in water absorption occurred after 35 days of modification, with a decrease of 41.48%, followed by 27.95% after 21 days of modification, and 20.92% after 14 days of modification. The reduction in water absorption can be attributed to the permeation of silicate materials from the permeable crystalline materials into the micro-cracks and voids of the old mortar, where it reacts with calcium hydroxide to generate C-S-H crystals that block the capillary channels. In addition, the silicate in the permeable crystalline materials adsorbs into the micropores of the old mortar, filling some of the pores and reducing the porosity [30,31]. The combined effect of these two factors leads to a decrease in water absorption of the recycled aggregates after modification, and an improvement in their performance.

### 3.3. Effect of Permeable Crystalline Materials on Workability of Recycled Aggregate Concrete

**Figure 7:**
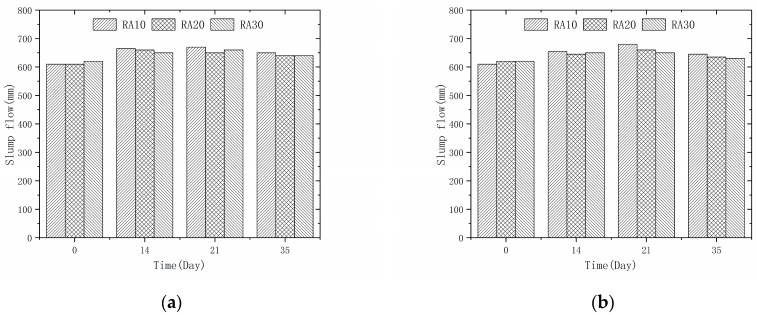
Recycled aggregate concrete slump flow: (**a**) 50% recycled aggregate replacement rate; (**b**) 100% recycled aggregate replacement rate.

**Figure 8:**
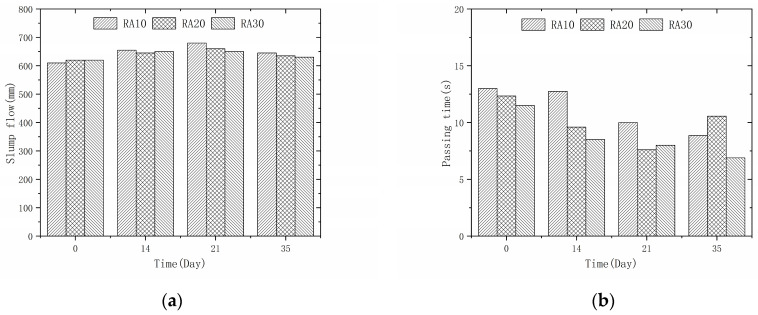
Recycled aggregate concrete V-funnel test time: (**a**) 50% recycled aggregate replacement rate; (**b**) 100% recycled aggregate replacement rate.

The slump flow (SF) and V-funnel flow time (VF) of recycled aggregate concrete modified by penetrating crystalline materials are presented in Figure 7 and Figure 8, respectively. The SF measures the flowability of the concrete and the VF measures its viscosity. The figures demonstrate that after one hour of saturation treatment of the recycled aggregate, the concrete meets the required flowability standard (SF > 600 mm, 5 s < VF < 25 s). Moreover, the rheological properties of the recycled aggregate concrete are further improved after modification with penetrating crystalline materials. The reduction in water absorption of the modified recycled aggregate decreases the amount of free water during the mixing process and increases the viscosity of the mixture, resulting in better rheological properties of the concrete. However, these properties are still inferior to those of natural aggregate concrete [32]. From Figure 8, it is evident that the expansion of the concrete gradually increases as the modification time increases from 0 to 21 days. This is because the reduction in water absorption of the aggregate increases the effective water-to-cement ratio of the recycled aggregate concrete. However, as the modification time increases from 21 to 35 days, the expansion of the concrete shows a decreasing trend. This is because more silicate is adsorbed on the surface of the recycled aggregate with longer modification times, which has a larger size than cement and a higher water absorption rate, thus reducing the slump flow of the recycled aggregate concrete.

### 3.4. Compressive Strength of Recycled Aggregate Concrete Modified by Permeable Crystalline Materials

#### 3.4.1. Effect of Replacement Rates of Recycled Aggregate on Compressive Strength

**Figure 9:**
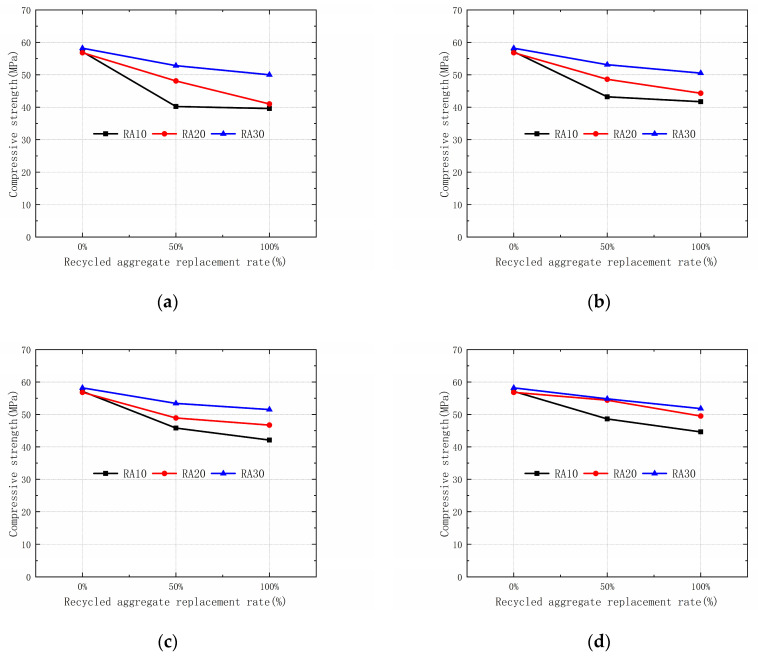
Compressive strengths of different replacement aggregate rates: (**a**) 0 day; (**b**) 14 day; (**c**) 21 day; (**d**) 35 day.

The 28-day compressive strength results of recycled aggregate concrete with varying replacement rates are presented in Figure 9. As the replacement rate of recycled aggregate increases, the 28-day compressive strength of recycled aggregate concrete decreases. When the recycled aggregate is unmodified and the replacement rate of the aggregate increases from 0% to 50%, the compressive strengths of the three types of recycled aggregate concrete decrease by 29.6%, 15.3%, and 9.27%, respectively. When the replacement rate of recycled aggregate increases from 0% to 100%, the compressive strengths of the three types of recycled aggregate concrete decrease by 30.64%, 27.8%, and 14.1%, respectively. The recycled aggregate concrete modified by permeable crystalline materials also shows a similar trend, with the compressive strength decreasing as the replacement rate of the aggregate increases. These results are consistent with the findings of previous studies on the compressive strength of recycled concrete cubes by Gao [33] and Xie [34].

#### 3.4.2. Effect of Initial Strength of Aggregates on Compressive Strength of Recycled Aggregate Concrete

**Figure 10:**
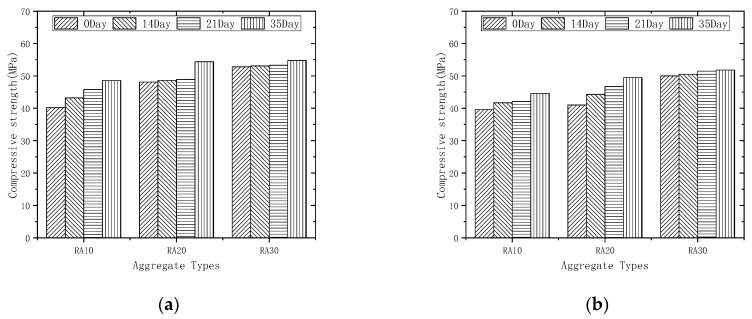
Compressive strengths of different aggregate initial strengths: (**a**) 50% recycled aggregate replacement rate; (**b**) 100% recycled aggregate replacement rate.

Figure 10 illustrates the correlation between the initial strength of recycled aggregates and the compressive strength of recycled aggregate concrete. The initial design strengths of the three types of aggregates are C10, C20, and C30. When the aggregate replacement rate is 50% and the aggregates are unmodified, RAC30 and RAC20 exhibit compressive strengths that are 31.34% and 19.65% higher than RAC10, respectively. This trend persists even after 14, 21, and 35 days of modification with permeable crystalline materials, with RAC20 having compressive strengths 6.77%, 11.93%, and 12.50% higher than RAC10, respectively, and RAC30 having compressive strengths 16.59%, 22.92%, and 12.76% higher than RAC10, respectively. A similar pattern is observed when the aggregate replacement rate is 100%.

#### 3.4.3. Effect of Modification Time on the Compressive Strength

**Figure 11:**
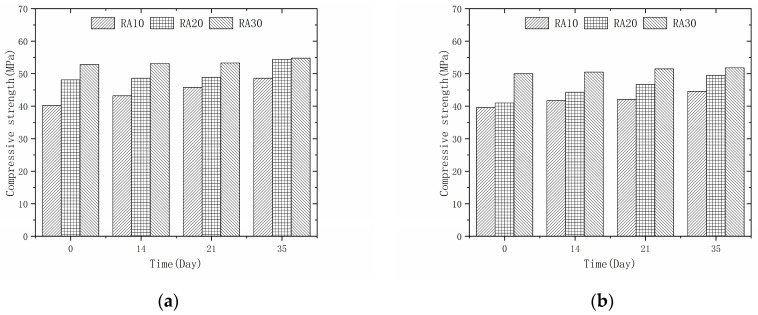
Compressive strengths of different modification times: (**a**) 50% recycled aggregate replacement rate; (**b**) 100% recycled aggregate replacement rate.

Figure 11 illustrates the relationship between the compressive strength of recycled aggregate concrete after 28 days and the duration of permeable crystalline materials modification. Generally, for a fixed replacement rate of recycled aggregate, the compressive strength of recycled aggregate concrete shows a continuous increase as the modification time of the permeable crystalline materials increases. At a substitution rate of 50% and after 14, 21, and 35 days of modification, the compressive strength of recycled aggregate concrete increased by 0.57% to 7.71%, 0.95% to 13.93%, and 3.79% to 20.90%, respectively. Similarly, for a replacement rate of 100%, the compressive strength of recycled aggregate concrete increased by 1.00% to 8.05%, 3.00% to 13.93%, and 3.79% to 20.73% after 14, 21, and 35 days of modification, respectively. The results indicate that the modified permeable crystalline materials have a strengthening effect on the compressive strength of recycled aggregate concrete to some extent. Additionally, for the same modification time and replacement rate of recycled aggregate, the increase in the compressive strength of recycled aggregate concrete is more significant for lower initial strength of the aggregate. This suggests that the modification effect of the permeable crystalline materials is better for low-strength recycled aggregate.

### 3.5. Effect of Permeable Crystalline Materials on the Pore Structure of Recycled Aggregate Concrete

The pore sizes can be classified into four categories according to Wu’s classification: more harmful pores (>200 nm), harmful pores (100–200 nm), less harmful pores (20–100 nm), and harmless pores (<20 nm) [35].

**Figure 12:**
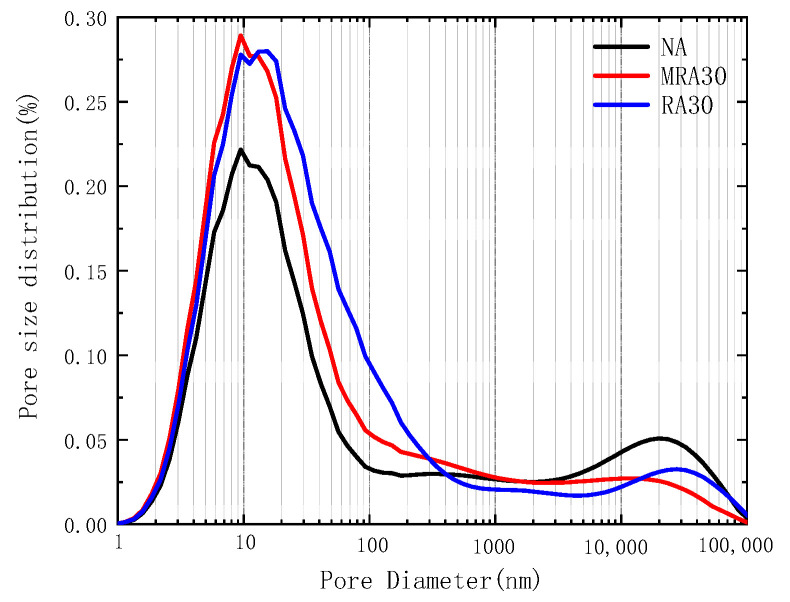
Recycled aggregate concrete pore size distribution.

Figure 12 illustrates the pore distribution of three types of concrete. The figure demonstrates that all three types of concrete display a significant increase and decrease between 1 nm and 100 nm, with peaks in their distributions, indicating that the primary pore size distribution of the three types of concrete falls within the 1–100 nm range. The order of the distribution peaks observed was natural aggregate concrete, enhanced recycled aggregate concrete, and recycled aggregate concrete, with the peak pore size increasing in that order. This suggests that the pore size distribution of concrete decreases after reinforcing with permeable crystalline materials. Once the peak is achieved, the distribution rate of pores in enhanced recycled aggregate concrete slows down as the pore size increases. This implies that the pore size distribution in the reinforced recycled aggregate concrete is more uniform, and the average pore size is reduced after reinforcing with permeable crystalline materials.

**Figure 13:**
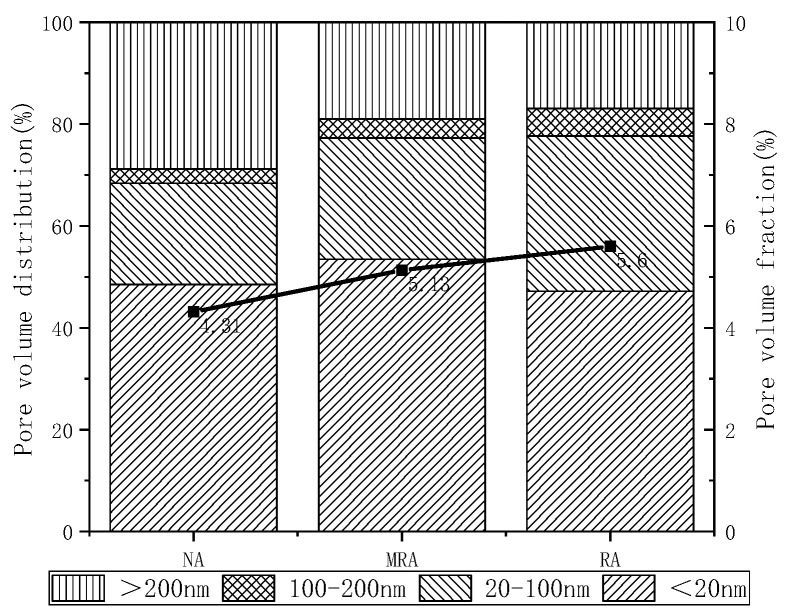
Recycled aggregate concrete pore volume distribution.

From Figure 13, it can be observed that the recycled aggregate concrete reinforced with permeable crystalline materials exhibits the most significant reduction in the percentage of fewer harmful pores compared to recycled aggregate concrete, decreasing from 30.48% to 23.85%, a decrease of 6.63%. The growth of harmless pores is most evident, increasing from 47.17% to 53.44%, an increase of 6.27%. There was a slight decrease in the percentage of multiple harmful pores, from 5.41% to 3.71%, a decrease of 1.70%. The pore size distribution shows a slight increase in the percentage of harmful pores, from 16.94% to 19.00%, an increase of 2.06%.

The use of permeable crystalline materials in the recycled aggregate concrete has been shown to reduce the percentage of harmful pores, including fewer harmful and harmful pores. The reinforcement process involves soaking the recycled aggregate and aged mortar in the permeable crystalline materials, which triggers a secondary hydration reaction and generates C-S-H crystals that fill larger voids, thereby breaking down large pores into smaller ones. The nano-silicon ions, which were not fully reacted, were directly dispersed into the concrete during mixing, promoting the concrete hydration reaction and filling the internal structure of the concrete. This results in a denser and better distributed pore structure in the reinforced recycled aggregate concrete.

### 3.6. Effect of Permeable Crystalline Materials on the Microstructure of Recycled Aggregate Concrete

The internal microstructures of recycled aggregate and recycled aggregate concrete were analyzed using SEM. Samples of recycled aggregates, modified recycled aggregates, and natural aggregates were selected for aggregate testing. For concrete testing, samples of modified recycled aggregate concrete, recycled aggregate concrete, and natural aggregate concrete were selected.

**Figure 14:**
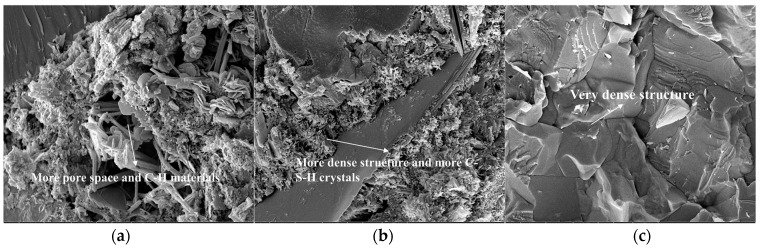
Microstructure and morphology of aggregate: (**a**) recycled aggregate; (**b**) modified recycled aggregate; (**c**) natural aggregate.

Figure 14 illustrates the microstructure of natural and recycled aggregate before and after modification with permeable crystalline materials, respectively. The figure shows that the modified recycled aggregate has a denser structure and fewer pores in the old mortar. The modification results in a significant amount of silica filling the cracks and pores of the aggregate, resulting in improved strength of the old mortar. Additionally, the reaction of silica with the old mortar generates crystals that fill the pores and cracks, thereby improving the performance of the aggregate.

**Figure 15:**
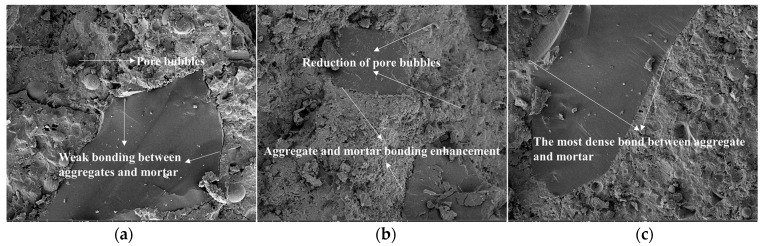
Microstructure and morphology of concrete: (**a**) recycled aggregate concrete; (**b**) modified recycled aggregate concrete; (**c**) natural aggregate concrete.

From Figure 15, it is evident that the unmodified recycled aggregate concrete exhibits significant cracking at the interface between the aggregate and mortar, while the mortar contains numerous pores and bubbles. However, the surface of the recycled aggregate concrete modified by the permeable crystalline materials adsorbs a substantial amount of silicate crystals, which undergo hydration reactions and filling effects during the concrete mixing process. As a result, the bonding strength between the aggregate and new mortar is enhanced, and the mortar contains fewer bubbles and pores. Notably, the structure of natural aggregate concrete is the most compact, and the bonding between the aggregate and mortar is the tightest.

**Figure 16:**
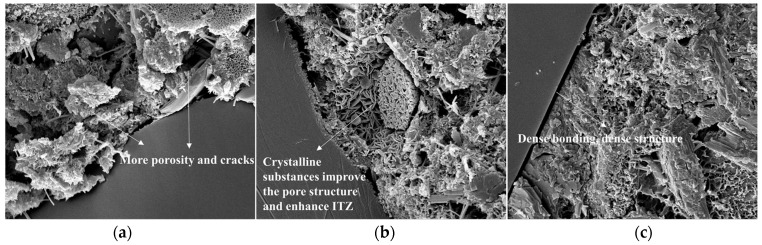
Microstructure and morphology of concrete: (**a**) recycled aggregate concrete; (**b**) modified recycled aggregate concrete; (**c**) natural aggregate concrete.

Figure 16 displays the SEM images of the three types of recycled aggregate concrete magnified 10,000 times. The unmodified recycled aggregate interface transition zone contains numerous pores and cracks, as depicted in the figure. The permeable crystalline materials’ silicates react with Ca(OH)_2_ and water in the concrete, promoting cement hydration and resulting in a denser concrete structure that enhances its performance. The mesh-like C-S-H crystal accumulations are plentiful, creating a denser structure that fills pores and cracks while strengthening the ITZ. Nevertheless, natural aggregate concrete has the most compact structure and highest ITZ strength compared to the other two types of concrete.

## 4. Discussion

### 4.1. Mechanistic Analysis of Recycled Aggregate Modified by Permeable Crystalline Materials

Improving the physical properties of recycled aggregate is crucial due to the presence of a significant number of pores and micro-cracks. The use of permeable crystalline materials to modify recycled aggregate can significantly enhance its physical properties. In this section, we establish a model to analyze the modification of recycled aggregate based on the improvement of both macroscopic and microscopic properties, thus revealing the mechanism of permeable crystalline materials modification of recycled aggregate.

**Figure 17:**
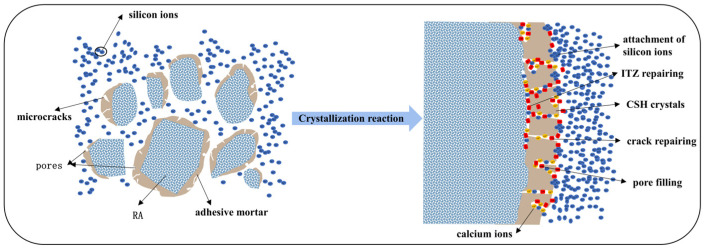
Aggregate modification model: Red represents CSH crystals; Orange represents calcium ions; Blue represents silicon ions.

**Figure 18:**
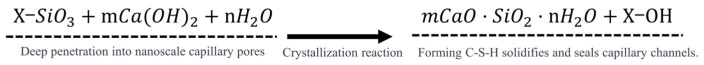
Permeable crystalline materials reaction model.

**Figure 19:**
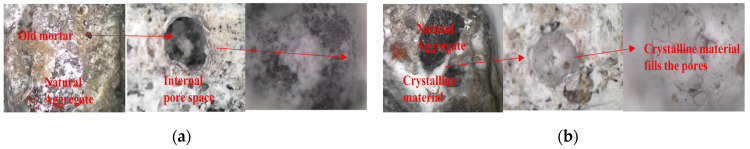
Surface condition of recycled aggregate: (**a**) recycled aggregate concrete; (**b**) modified recycled aggregate concrete.

**Figure 20:**
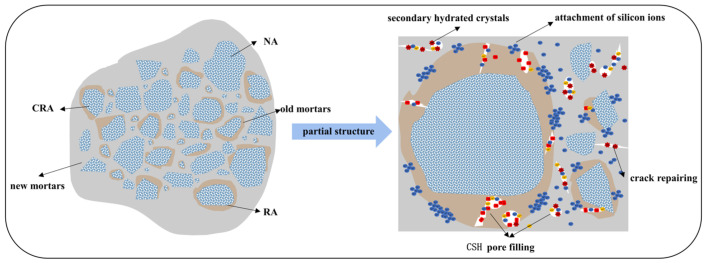
Recycled aggregate concrete modification model: Red represents CSH crystals; Orange represents calcium ions; Blue represents silicon ions.

The reaction mechanism of permeable crystalline material-enhanced recycled aggregate is presented in Figure 17 and Figure 18. Additionally, Figure 19 shows the surface of the modified recycled aggregate, observed using a high-magnification electron microscope. After modifying the aggregate with permeable crystalline materials, a chemical reaction occurs between the calcium ions in the mortar and the modified material, resulting in the generation of C-S-H crystals. These crystals fill the internal pores and micro-cracks of the mortar, which inhibits crack formation and reduces porosity. As a result, this process enhances the density of the recycled aggregate structure. Moreover, Figure 20 shows that unreacted sodium silicate directly fills the pores and micro-cracks of the old mortar, reducing the volume of larger pores and micro-cracks and decreasing the porosity of the recycled aggregate. Both effects combined result in the improvement of the pore structure of the recycled aggregate, leading to a more compact structure, reduced water absorption, and a lower crushing index. Consequently, the physical properties of the recycled aggregate are improved.

### 4.2. Mechanistic Analysis of Recycled Aggregate Concrete Modified by Permeable Crystalline Materials

The performance of recycled aggregate concrete (RAC) can be improved by modifying it with infiltrating crystallization material, as depicted in the mechanism diagram:

(1) The water absorption and crushing index of the modified recycled aggregate are both improved. When mixed with new concrete, the reduced water absorption of the modified recycled aggregate leads to a decrease in its water content in the new concrete, resulting in less evaporation of hydration water and fewer formed pores, resulting in a denser structure. The decrease in the crushing index of the modified recycled aggregate leads to a higher interfacial transition zone (ITZ) strength in the new concrete, thereby improving the performance of the modified RAC.
(2) After long-term soaking with sodium silicate, a large amount of unreacted sodium silicate adheres to the surface of the recycled aggregate. The sodium silicate adhering to the surface of the recycled aggregate disperses with the cement to various parts of the concrete during the mixing process. The sodium silicate promotes the hydration reaction of the cement, making the hydration reaction of the concrete more complete and the structure denser, thereby improving the performance of the recycled aggregate concrete.

### 4.3. Analysis of Economic and Carbon Emissions

According to the survey, the current market price of natural aggregates is 85 RMB per ton and recycled aggregates are 50 RMB per ton. The cost of permeation crystallization material needed to process one ton of recycled aggregate is 15 RMB. With the same use of other materials, the use of permeation crystallization material to modify the recycled aggregate for concrete precast can directly bring an economic benefit of 20 RMB per ton.

According to the specification (calculation method and evaluation standard of carbon emission from concrete), the CO_2_ emissions from concrete production are given by Equation (1):

(1) $$G_{1}=\sum Q_{i}\times EF_{i}$$

where $G_{1}$ is the CO_2_ emissions from the production process, $Q_{i}$ is the consumption of raw material type *i*, and $EF_{i}$ is the CO_2_ emissions from the production process of raw material type *i*. The detailed data are shown in Table 4.

According to the concrete mix design, when using recycled aggregates instead of all natural aggregates to prepare concrete, 1 ton of recycled aggregate concrete can reduce 3.98 kg of CO_2_ emissions. The use of recycled aggregates can greatly reduce the extraction of natural sand and gravel, which not only brings huge economic benefits, but also greatly reduces carbon dioxide emissions. Overall, enhancing the use of recycled aggregates can significantly improve economic efficiency and reduce CO_2_ emissions.

## 5. Conclusions

This study investigates the mechanical properties and microstructure of recycled aggregate (RA) and recycled aggregate concrete (RAC) modified by permeable crystalline materials. Two experimental stages were conducted. In the first stage, different initial strength aggregates were immersed in the permeable crystalline materials for varying periods to analyze the modification effect of the recycled aggregate, and the enhancement of compressive strength in RAC was studied under different substitution rates. In the second stage, nuclear magnetic resonance and SEM tests were conducted on recycled aggregate and RAC to analyze the effect of permeable crystalline materials on their pore distribution and interfacial transition zone and to study the multiscale properties of permeable crystalline materials-modified RAC. The study concludes that:

(1) The crushing index and water absorption rate of recycled aggregate decrease with increasing modification time using permeable crystalline materials. However, the rate of decrease slows down as the modification time increases.
(2) For recycled aggregate concrete (RAC) with the same initial strength and substitution rate of aggregate, the compressive strength increases with increasing modification time. However, for RAC with the same initial strength and modification time, the compressive strength decreases with an increasing substitution rate of aggregate. Additionally, under a consistent modification time and substitution rate, the compressive strength of RAC increases with increasing initial strength of the aggregate.
(3) The rheological properties of recycled aggregate self-compacting concrete were improved after modification with permeable crystalline materials. When the recycled aggregate was modified for too long, the concrete rheological properties decreased instead. Due to the excessive silicate adhering to the surface of recycled aggregate, the effective water–cement ratio of concrete decreases and the rheological performance decreases.
(4) The RAC modified with permeable crystalline materials exhibits improved compressive strength. This is attributed to the formation of C-S-H crystals through the reaction between the silicate and calcium hydroxide in the material, which seals capillary channels and fills pores, thereby increasing the strength of recycled aggregate and reducing water absorption rates. Moreover, during the mixing process, the penetrating crystalline materials fall off the aggregate surface, filling the pores and cracks in the concrete and increasing its compactness.
(5) The performances of recycled aggregates and recycled aggregate concrete are enhanced at multiple scales through the use of permeable crystalline materials, as demonstrated through microscopic and mechanistic analysis. This approach simultaneously improves the properties of recycled aggregates and recycled aggregate concrete.

## Figures and Tables

**Table 1:** Chemical composition and physical properties of the materials.

Material	CaO	SiO_2_	Al_2_O_3_	MgO	SO_3_	Fe_2_O_3_	K_2_O	P_2_O_5_	TiO_2_
Cement	69.32	16.50	3.61	1.24	3.08	4.06	1.28	0.24	0.44
FA	37.32	30.60	7.58	2.46	4.92	14.60	0.58	1.22	0.88

**Table 2:** Physical properties of aggregates.

Aggregate Types	RA10	RA20	RA30	NA
Water absorption (%)	4.35	3.66	2.29	0.55
Crushing index (%)	25.71	22.74	20.82	13.21

**Table 3:** Mix proportion of concrete.

Group	Mixture	C	W	S	NA	RA	CRA	FA	SP
1	NAC	390	188	820	824			133	2.2
2	RAC10–0–50%	390	188	820	412	412		133	2.2
3	RAC10–0–100%	390	188	820		824		133	2.2
4	MRAC10–14–50%	390	188	820	412		412	133	2.2
5	MRAC10–14–100%	390	188	820			824	133	2.2
6	MRAC10–21–50%	390	188	820	412	412		133	2.2
7	MRAC10–21–100%	390	188	820		824		133	2.2
8	MRAC10–35–50%	390	188	820	412		412	133	2.2
9	MRAC10–35–100%	390	188	820			824	133	2.2
10	RAC20–0–50%	390	188	820	412	412		133	2.2
11	RAC20–0–100%	390	188	820		824		133	2.2
12	MRAC20–14–50%	390	188	820	412		412	133	2.2
13	MRAC20–14–100%	390	188	820			824	133	2.2
14	MRAC20–21–50%	390	188	820	412		412	133	2.2
15	MRAC20–21–100%	390	188	820			824	133	2.2
16	MRAC20–35–50%	390	188	820	412		412	133	2.2
17	MRAC20–35–100%	390	188	820			824	133	2.2
18	RAC30–0–50%	390	188	820	412		412	133	2.2
19	RAC30–0–100%	390	188	820			824	133	2.2
20	MRAC30–14–50%	390	188	820	412		412	133	2.2
21	MRAC30–14–100%	390	188	820			824	133	2.2
22	MRAC30–21–50%	390	188	820	412		412	133	2.2
23	MRAC30–21–100%	390	188	820			824	133	2.2
24	MRAC30–35–50%	390	188	820	412		412	133	2.2
25	MRAC30–35–100%	390	188	820			824	133	2.2

Notes: RAC30–0–100% indicates that RA30 aggregate is 100% substituted for natural aggregate in the preparation of recycled aggregate concrete; MRAC30–35–50% indicates modified recycled aggregate concrete prepared by replacing 50% of natural aggregates with 35 days of modified RA30 aggregates.

**Table 4:** CO_2_ emissions per unit of raw material production process.

No.	Raw Material Variety	Unit of Measure	CO_2_ Emissions (kgCO_2_/kg)
1	Cement	kg	0.732
2	Slag	kg	0.0624
3	Fly ash	kg	0.0345
4	Natural sand	kg	0.00398
5	Mechanical sand	kg	0.0417
6	Recycled aggregate	kg	0
7	Natural aggregate	kg	0.00398
8	Additive	kg	0.72
9	Water	kg	0.000148
10	Others	kg	0.0442

## Data Availability

No new data were created or analyzed in this study. Data sharing is not applicable to this article.
